# Quality Assessment of Large Language Model–Generated Medical Dialogue for Clinical Vignettes: Evaluation Study

**DOI:** 10.2196/80752

**Published:** 2025-11-03

**Authors:** Yasutaka Yanagita, Daiki Yokokawa, Shiichi Ihara, Ryo Yoshida, Yoshihide Okano, Takanori Uehara

**Affiliations:** 1 Department of General Medicine Chiba University Hospital, Chiba, Japan Chiba Japan

**Keywords:** ChatGPT, clinical vignettes, artificial intelligence, generative AI, medical education, physician-patient dialogue

## Abstract

**Background:**

Traditional clinical vignettes, though widely used in medical education, often focus on prototypical presentations; require substantial time and effort to develop; and fail to represent patient diversity, the complexity of clinical conditions, patients’ perspectives, and the dynamic nature of physician-patient interactions.

**Objective:**

This study aimed to evaluate the quality of Japanese-language physician-patient dialogues produced by generative artificial intelligence (AI), focusing on their medical accuracy and overall appropriateness as medical interviews.

**Methods:**

We created an AI prompt that included a specific clinical history and instructed the model to simulate a cooperative patient responding to the physician’s questions to generate a physician-patient dialogue. The target diseases were those covered by the Japanese National Medical Licensing Examination. Each dialogue consisted of 25 turns by the physician and 25 by the patient, reflecting the typical volume of conversation in Japanese outpatient settings. Three internists independently evaluated each generated dialogue using a 7-point Likert scale across 6 criteria: coherence of the conversation, medical accuracy of the patient’s responses, medical accuracy of the physician’s responses, content of the medical history, communication skills, and professionalism. In addition, a composite score for each dialogue was calculated as the overall mean of these 6 criteria. Each dialogue was also examined for the presence of 5 essential clinical components commonly included in medical interviews: chief concern and clinical course since onset, physical findings, test results, diagnosis, and treatment course. A dialogue was considered to include a component only if all 3 evaluators independently confirmed its presence.

**Results:**

The mean composite score was 5.7 (SD 1.0), indicating high overall quality. Mean scores for each criterion were as follows: coherence of the conversation, 5.9 (SD 0.9); medical accuracy of the patient’s responses, 6.0 (SD 0.9); medical accuracy of the physician’s responses, 5.6 (SD 1.1); content of medical history taking, 5.9 (SD 0.9); communication skills, 5.6 (SD 0.9); and professionalism, 5.5 (SD 1.1). Among the 5 clinical components assessed in each dialogue across 47 clinical cases, chief concern and clinical course were included in all 47 (100%) cases, physical findings in 15 (32%) cases, test results in 27 (57%) cases, diagnosis in 45 (96%) cases, and treatment course in 0 (0%) cases.

**Conclusions:**

While physician oversight remains essential, it is feasible to efficiently create AI-generated educational materials for medical education that overcome the limitations of traditional clinical vignettes. This approach may reduce time and financial burdens, enhancing opportunities to practice clinical interviewing in settings that closely mirror real-world encounters.

## Introduction

Artificial intelligence (AI) is critical for the advancement of medicine. In particular, generative AI, exemplified by large language models (LLMs), has the potential to fundamentally transform health care. Tools such as ChatGPT, developed by OpenAI, possess advanced conversational capabilities and broad applicability [1], with an increasing number of applications in the medical field. From the perspective of conversational capabilities, generative AI–based chatbots can provide rehabilitation guidance and mental health support to patients and assist health care professionals in patient management [2]. Generative AI is versatile enough to have passed the Japanese National Medical Licensing Examination [3]. Moreover, LLMS are now used to enter patients’ medical histories and verify diagnostic accuracy in English and Japanese [4,5], indicating that the scope of generative AI applications is expected to expand further in the future.

The application of generative AI in medical education has attracted increasing interest. The use of generative AI to produce physician-patient dialogues has the potential to replicate disease-specific communication encountered in clinical practice and serve as a valuable educational resource for medical students.

A comparable and widely used educational tool in medical education research is the *clinical vignette* [6-8]. Vignettes are paper-based instructional resources that present a patient’s medical history and key clinical information in an organized written format. They are considered highly effective in helping medical students understand disease concepts, symptomatology, and treatment pathways [9].

Exposure to various vignettes can deepen students’ clinical understanding. However, traditional vignettes often lack diversity in case scenarios and tend to focus narrowly on diagnostic information, thereby failing to capture the complexity, ambiguity, and uncertainty that characterize real-world clinical practice [10]. In addition, the development of vignettes requires substantial time and effort. As clinical vignettes are generally written from the health care provider’s perspective, they inherently fail to capture the patient’s viewpoint and the dynamics of physician-patient interaction. Using generative AI such as ChatGPT to create dialogue-based educational materials that incorporate these missing elements is considered a promising approach. These educational materials offer several advantages. First, ChatGPT can generate a wide variety of dialogues tailored to different diseases and clinical scenarios, enabling repeated practice. Its use can reduce both the time and financial costs associated with producing educational content. Moreover, medical students can engage in clinical reasoning by organizing information while considering multiple differential diagnoses and can practice effective physician-patient communication, as would be required in actual clinical settings.

AI-generated dialogues incorporate the medical information necessary for diagnostic decision-making along with a variety of interactions that closely resemble those encountered in real clinical settings. This enables medical students to develop information-processing skills in authentic, context-rich scenarios. Through this process, students are expected to acquire the ability to identify clinically relevant information and develop a broader range of clinical competencies, including medical interviewing techniques, patient management skills, and effective communication strategies.

This study examined the feasibility of generating physician-patient dialogues using generative AI and evaluated the quality of the generated dialogues. Generative AI has already demonstrated the ability to generate differential diagnosis lists, illness scripts, and clinical vignettes based on medical information [11,12]. Accordingly, it is considered feasible to generate physician-patient dialogues with a high level of accuracy using generative AI.

In this study, we assessed the quality of dialogues generated in Japanese medical interviews, including their medical accuracy, and investigated their potential utility as educational materials in the context of medical education. By demonstrating the feasibility of easily generating diverse physician-patient dialogues using generative AI, it becomes possible to efficiently produce a large volume of educational content for use in medical education under the supervision of physicians with medical expertise. The appropriate application of AI technology is expected to increase the opportunities for medical students to engage with more practical and interactive content, thereby enhancing the effectiveness of medical education.

## Methods

### Overview

Physician-patient dialogues were generated using an LLM and were subjected to cross-sectional evaluation. To ensure relevance to medical education, 47 target diseases were selected to represent diseases that medical students were expected to learn. The selection was based on the content of the Japanese National Medical Licensing Examination [13] and finalized through discussion between a board-certified internist (YY) and a board-certified family physician (DY; Textbox 1). To evaluate the generated dialogues, we recruited 3 Japanese internists (SI, RY, and YO) who are actively involved in diagnostic practice at a university hospital and regularly manage a wide range of diagnostically challenging cases. These internists assessed the quality of the dialogues.

**List of 47 diseases used to generate and evaluate artificial intelligence–generated physician-patient dialogues.**

**Diseases**

1. Gastroesophageal reflux disease
2. Functional dyspepsia
3. Esophageal achalasia
4. Crohn disease
5. Appendicitis
6. Pheochromocytoma
7. Primary aldosteronism
8. Cushing syndrome
9. Hashimoto disease
10. Subacute thyroiditis
11. Graves disease
12. Depression
13. Panic disorder
14. Gout
15. Pneumothorax
16. Myasthenia gravis
17. Trigeminal neuralgia
18. Parkinson disease
19. Alzheimer disease
20. Benign paroxysmal positional vertigo
21. Migraine
22. Cluster headache
23. Neurocardiogenic syncope
24. Intervertebral disk herniation
25. Insomnia
26. Bronchial asthma
27. Acute eosinophilic pneumonia
28. Rheumatoid arthritis
29. Systemic lupus erythematosus
30. Sjögren syndrome
31. Measles
32. Fibromyalgia
33. Gallstone attack
34. Acute cholecystitis
35. Unstable angina
36. COVID-19
37. Lumbar spinal stenosis
38. Thromboangiitis obliterans
39. Infectious mononucleosis
40. Streptococcal pharyngitis
41. Hepatitis
42. Transient ischemic attack
43. Iron deficiency anemia
44. Heatstroke
45. Acute pericarditis
46. Chronic obstructive pulmonary disease
47. Lung cancer

### Production Environment

The analysis was performed on a terminal running Ubuntu (version 20.04.2 Long-Term Support; Canonical). The central processing unit was equipped with an AMD EPYC 7402*P* 24-core processor (Advanced Micro Devices, Inc), with 256 GB of main memory. An NVIDIA A100 graphics processing unit (NVIDIA Corp) with 40 GB of RAM was used for the computations. Python (version 3.6.9) and the OpenAI Python library (version 0.27.8; OpenAI) were used. The application programming interface used to generate the dialogues was the latest one available at the start of the study, gpt-4o-2024-11-20 [14], and the dialogues were generated on November 22, 2024.

### Prompts to Be Entered Into the LLM

**Figure 1:**
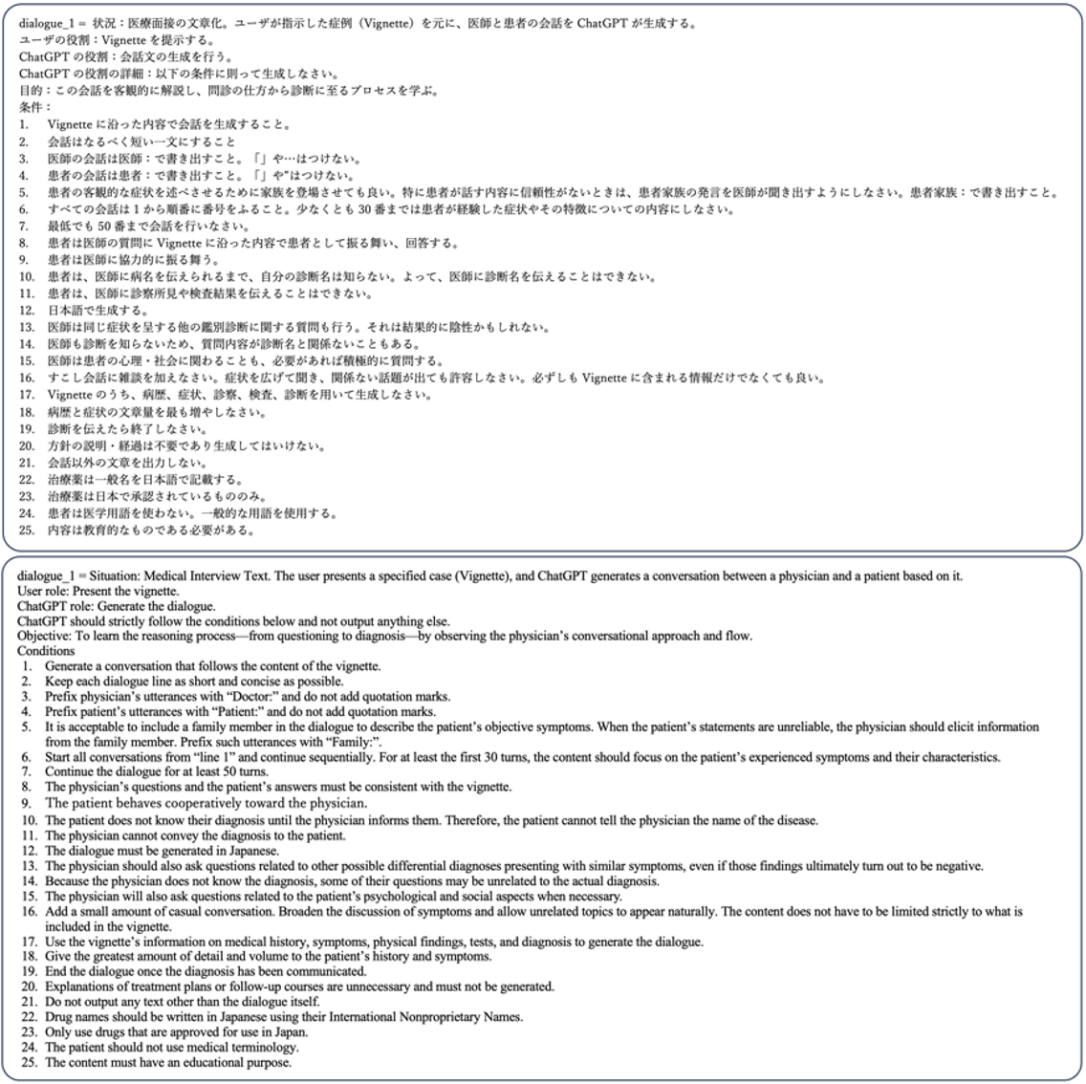
A portion of the prompt used to generate physician-patient dialogues in Japanese using generative artificial intelligence (Top: Japanese, Bottom: English).

Referring to previous studies that used AI to generate vignettes and illness scripts [12], concise instructions specifying the desired output conditions were provided to the LLM as prompts (Figure 1). To enhance the completeness and coherence of the generated dialogues, the prompts included conditions such as the user supplying a specific vignette before the generation, refraining from using medical terminology, and responding cooperatively to the physician’s questions. The dialogue length was determined with reference to the average volume of conversation in typical outpatient consultations (approximately 5-10 min) [15], and the LLM was instructed to generate 25 turns each from the physician and the patient.

### Evaluation of Dialogue

The presence of five key components commonly included in medical interviews was assessed: (1) chief concern and clinical course since onset, (2) physical findings, (3) test results, (4) diagnosis, and (5) treatment course. Three physicians independently reviewed each dialogue, and a dialogue was considered to contain all 5 components only if all 3 physicians confirmed their inclusion.

Next, criteria for assessing the quality of the medical interviews were developed. Six evaluation criteria were selected through discussions between a board-certified internist (YY) and a board-certified family physician (DY), with reference to the evaluation domains of the Mini-Clinical Evaluation Exercise (Mini-CEX) [16]: (1) coherence of the conversation, (2) medical accuracy of the patient’s statements, (3) medical accuracy of the physician’s statements, (4) quality of the physician’s history taking, (5) communication skills, and (6) professionalism.

Criterion 1, coherence of the conversation, was evaluated as a linguistic criterion, focusing on the smoothness of the interaction between the physician and patient, the presence of inconsistencies, grammatical or typographical errors, and the logical relationship of the dialogue. Criteria 2 and 3, medical accuracy of the patient’s and physician’s statements, respectively, were assessed as clinical criteria by evaluating their consistency with the known clinical features of the respective diseases. Criterion 4, history taking, was assessed based on whether the physician elicited information regarding symptom characteristics and exacerbating or relieving factors. Criteria 5 and 6, communication skills and professionalism, respectively, were evaluated by assessing whether the physician explored the patient’s explanatory model and demonstrated respect, compassion, and empathy toward the patient (Table 1).

Following prior studies [12], we specifically employed 3 Japanese physicians affiliated with a university hospital, each of whom was involved in the supervision and education of medical students and residents, to evaluate the generated dialogues. Each of the 6 evaluation criteria was rated on a 7-point Likert scale, based on the perceived educational usefulness of the medical students. The scale was defined as follows: 1=not applicable at all or not useful—major overall revision required, 2=low usefulness—multiple major revisions needed, 3=limited usefulness—some valuable content but substantial revisions necessary, 4=moderate usefulness—both strengths and several areas for improvement, 5=generally useful—some revisions or adjustments desirable, 6=high usefulness—only minor adjustments possibly needed, and 7=extremely useful and complete—no further revisions required.

For each dialogue, the score of each evaluation criterion was calculated as the average of the ratings of the 3 evaluators. The composite score for the dialogue was then derived by averaging the scores across all 6 evaluation criteria and interpreted using the same 7-point Likert scale.

**Table 1:** Evaluation criteria and definitions used to assess artificial intelligence–generated physician-patient dialogues in medical interviews.

Evaluation criteria	Evaluation details
Coherence of the conversation	Assessed whether the dialogue between the physician and patient proceeded smoothly, with accurate grammar, no typographical or spelling errors, and overall linguistic clarity
Medical accuracy of the patient’s statements	Evaluated whether the patient’s utterances accurately reflected the typical onset patterns, symptoms, and clinical course associated with the relevant disease
Medical accuracy of the physician’s statements	Assessed whether the physician’s explanations and other statements were medically accurate and aligned with established clinical knowledge
Quality of the physician’s history taking	Evaluated whether the physician asked about essential elements of the current illness, including symptom location, characteristics, severity, temporal course, contextual factors, aggravating and relieving factors, associated symptoms, and the patient’s response to the symptoms
Communication skills	Assessed whether the physician conducted the interview in a way that facilitated open communication, explored the patient’s explanatory model and psychosocial context, and confirmed the patient’s understanding of the information discussed
Professionalism	Evaluated whether the physician demonstrated respect, compassion, and empathy toward the patient and whether a trusting therapeutic relationship was established

### Ethical Considerations

This study did not involve human or animal participants, and therefore, ethics approval was not required.

## Results

Using the gpt-4o-2024-11-20 model, physician-patient dialogues were generated for 47 clinical cases (Table 2). Among the 47 generated dialogues, clinical component 1, chief concern and clinical course since onset, was present in all 47 (100%) cases; clinical component 4, diagnosis, was included in 45 (96%) cases, and in each of these cases, the model accurately outputted the specified disease name, as instructed. In contrast, clinical component 2, physical findings, was included in 15 (32%) cases; clinical component 3, test results, was included in 27 (58%) cases; and clinical component 5, treatment course, was not included in any of the cases (0%). Regarding the quality of the medical interviews, the average score was 5.9 (SD 0.9) for coherence of the conversation, 6.0 (SD 0.9) for medical accuracy of the patient’s statements, and 5.6 (SD 1.1) for medical accuracy of the physician’s statements. The average score was 5.9 (SD 0.9) for quality of the physician’s history taking, 5.6 (SD 0.9) for communication skills, and 5.5 (SD 1.1) for professionalism. The overall composite score, calculated as the mean of the 6 evaluation criteria, was 5.7 (SD 1.0).

A focused discussion was conducted among 5 physicians, 2 specialists (YY and DY), and 3 evaluators (SI, RY, and YO), centered on dialogues that were subject to point deductions to identify and clarify the specific issues present in the lower-rated dialogues. The results of this analysis are summarized in Table 3. For reference, one dialogue with a perfect average score of 7 and another that received a lower average score of 4 are provided in Multimedia Appendix 1 and Multimedia Appendix 2, respectively.

**Table 2:** Average scores for each of the 6 evaluation criteria used to assess artificial intelligence–generated physician-patient dialogues.

Evaluation criteria	Average score (SD)
Coherence of the conversation	5.9 (0.9)
Medical accuracy of the patient’s statements	6.0 (0.9)
Medical accuracy of the physician’s statements	5.6 (1.1)
Quality of the physician’s history taking	5.9 (0.9)
Communication skills	5.6 (0.9)
Professionalism	5.5 (1.1)

**Table 3:** Problems identified for each evaluation criterion based on expert review of lower-rated artificial intelligence–generated physician-patient dialogues.

Evaluation criteria	Problems
Coherence of the conversation	Responses to patient questions were omitted. Although some expressions were unnatural, overall coherence was maintained.
Medical accuracy of the patient’s statements	Typical responses regarding aggravating and relieving factors were not provided.
Medical accuracy of the physician’s statements	A diagnosis was rendered, despite the interview being insufficient. Diagnoses were finalized based on test results that were never mentioned as having been performed, indicating inappropriate or unjustified diagnostic reasoning. The response is that it cures a disease that cannot be cured.
Quality of the physician’s history taking	Redundant questions were asked regarding information that had already been provided. When multiple symptoms were present, it was unclear which symptom’s aggravating or relieving factors were being discussed. The structure and content of the medical interview were inadequate. Additional questioning was conducted about symptoms not initially reported by the patient. Unnecessary blood tests were included and described without justification.
Communication skills	No attempts were made to confirm the patient’s understanding of the information provided. The patient’s explanatory model was rarely elicited. The patient’s response lacked logical progression or flow.
Professionalism	No appropriate responses were provided following expressions of anxiety. The overall focus of the dialogue was limited to diagnostic questioning, with few utterances directed toward building rapport or fostering a therapeutic relationship.

## Discussion

### Principal Findings

This study aimed to generate physician-patient dialogue using generative AI and evaluate the prompt designs, the resulting outputs, and the overall quality of the dialogues. We created 47 dialogues based on diseases from the Japanese National Medical Licensing Examination and evaluated them using 6 criteria related to clinical communication. The dialogues consistently included the chief concern and clinical course and demonstrated high medical accuracy and coherence, while areas such as professionalism and inclusion of treatment information were less consistently addressed. The overall composite score was 5.7 (SD 1.0), indicating general usefulness with minor revisions required.

The analysis first focused on the outputs generated for 47 clinical cases and the corresponding prompt designs that produced them. Examination of the medical content essential for a clinical interview revealed that all dialogues included descriptions of the chief concern and clinical course since onset and that accurate diagnostic labels were presented in 45 (96%) of the 47 cases. In contrast, none of the dialogues included information regarding the treatment course. In designing the prompts, the number of dialogue turns was set at 25 for each participant (50 in total), considering the average consultation time in Japanese outpatient settings, which is approximately 5 minutes. As an exploratory extension, we tested longer dialogues (50 and 100 turns per speaker). Although brief mentions of treatment began to appear, these dialogues became increasingly verbose, with redundant differential diagnoses and questioning. This indicated a decline in dialogue quality and educational effectiveness, highlighting the need to identify an optimal dialogue length in prompt design. Although the generation of information related to the treatment course may be improved through more sophisticated prompt engineering [17,18] or future updates to generative AI models [19], prior studies have demonstrated that diagnostic reasoning and treatment management involve fundamentally distinct cognitive processes [20]. Therefore, separating these processes into distinct dialogue scenarios may be more educationally effective than integrating them into a single prompt.

The quality of the generated dialogues, produced in Japanese, was evaluated based on 6 criteria. Regarding criterion 1, coherence of the conversation, the dialogues were generally smooth and grammatically correct. This reflects the high-level natural language processing capabilities of generative AI and suggests its potential to replicate physician-patient interactions at a basic level of linguistic fidelity within the context of Japanese-language medical interviews. Regarding criterion 2, medical accuracy of the patient’s statements, although there were instances in which the patient did not provide clear responses to the physician’s questions, particularly in certain disease contexts, such ambiguity may reflect the nature of real-world clinical encounters. From an educational perspective, these instances may be valuable for simulating authentic dialogue dynamics. Criterion 3, medical accuracy of the physician’s statements, received comparatively lower ratings than other criteria. This may be attributable to the perceived need for greater domain-specific precision, particularly when formulating clinical questions and providing medically accurate explanations to patients. This finding aligns with previous research on the limitations of generative AI in clinical reasoning [12]. Regarding criterion 4, quality of the physician’s history taking, in actual clinical practice, once the probability of a particular disease increases based on the patient’s narrative, physicians typically engage in further in-depth inquiry. Generative AI appears to struggle with appropriately weighing clinical information and identifying which elements warrant further exploration; thus, at present, AI may be limited in its capacity to conduct history taking guided by probabilistic diagnostic reasoning. Regarding criterion 5, communication skills, the dialogues showed limited use of verbal cues such as acknowledgments or responses that convey an understanding of the patient’s statements, and expressions of empathy toward patients’ concerns were insufficient. In addition, there were a few attempts to elicit a patient’s explanatory model, which is a critical step toward building rapport. Prompt design that encourages empathic and interactive responses may help address these limitations in future development. Regarding criterion 6, professionalism, although no ethically inappropriate expressions were identified, the dialogues generally lacked explicit demonstrations of patient-centered attitudes, respect, or empathic concern.

It is important to note that, in face-to-face medical interviews, nonverbal cues, such as facial expressions and nodding, play a substantially role in promoting patient satisfaction and emotional attunement [21,22], and the absence of such elements constitutes a fundamental limitation of text-based dialogue evaluations. Moreover, a small number of dialogues contained clinically inappropriate content, such as failure to provide justification for a diagnosis or incorrect assertion that a typically incurable disease could be cured. However, no ethically problematic or professionally inappropriate statements were identified in the dataset. Taken together, these results, along with the overall composite score of 5.7 (SD 1.0), suggest that, while physician oversight and revision are essential, generative AI–based dialogues may serve as a valuable educational resource for teaching clinical communication skills to medical students and early-stage trainees.

Because of their underlying architecture, LLMs exhibit inherent output variability, and identical prompts do not consistently yield identical responses. Although this randomness poses challenges in terms of reproducibility and control, it offers opportunities for prompt-based modulation, whereby carefully designed prompts can elicit diverse and contextually appropriate outputs [23]. In this respect, the ability to generate a wide range of physician-patient dialogues represents a particularly compelling and pedagogically valuable feature.

In this study, to facilitate the acquisition of fundamental structures in history taking, the AI was instructed to assume the role of a cooperative patient who provided clear and responsive answers. However, the simulated patient did not need to be restricted to cooperative profiles. It is also feasible to generate dialogues featuring patients with diverse communicative behaviors, such as those who are angry, uncommunicative, or unable to articulate clearly because of their underlying health conditions.

On the basis of our findings, we suggest that physician-patient dialogues generated by generative AI can be developed into high-quality educational materials with relatively minimal effort, provided that particular attention is paid to the medical accuracy of the physician’s utterances and that supervising physicians revise the content as necessary. Given the capability of generative AI to rapidly produce medically relevant content, its application in medical education and clinical practice is expected to expand further in the coming years [24,25]. As demonstrated in previous studies, when generative AI is used in medical education [26], the ability of instructors to review and modify the generated content enables its use as a supplementary instructional resource. Considering the capacity of the model to generate dialogues tailored to a wide range of clinical scenarios, this approach, when used with appropriate oversight, could offer a highly flexible and scalable educational tool.

### Limitations

This study had 3 primary limitations. First, the version of the generative AI used in this study was gpt-4o-2024-11-20, and the evaluation was based on the outputs generated on November 13, 2024. As the performance of generative AI models may evolve with future updates, periodic reevaluation is necessary. Moreover, as multiple LLMs are available, the quality and characteristics of dialogues generated by other models may differ from those evaluated in this study.

Second, there is currently no standardized method for prompt construction when interacting with generative AI, and the content of the input prompt can significantly influence the quality and nature of the output. In this study, the extent to which variations in the input conditions affect the generated dialogues was not examined systematically. Further investigations are warranted to clarify the impact of prompt design on output quality.

Third, this study was conducted in Japanese, and all prompts and generated dialogues were also in Japanese. Although prior research suggests a strong potential for adaptation to other languages, further validation is warranted.

### Conclusions

In this study, physician-patient dialogues were generated using an LLM, that is, generative AI. These findings indicate that, although physician supervision remains essential, such dialogues can be easily developed into materials suitable for use in medical education. In particular, dialogues that incorporate the patient’s perspective and the interactive elements of physician-patient communication could provide practical learning experiences that are difficult to achieve with conventional educational resources, suggesting a wide range of possible applications in medical education.

Moreover, the use of generative AI enables the efficient and large-scale creation of diverse educational content. This represents a significant advantage in terms of reducing the substantial time and effort traditionally required to develop medical instructional materials. By using this approach, medical students are expected to have more opportunities to learn in contexts that closely resemble actual clinical practice, thereby facilitating the acquisition of more practical medical interview skills.

## Appendix

Multimedia Appendix 1 Dialogue with a perfect average score (case 21: migraine).

Multimedia Appendix 2 Dialogue with a lower average score of 4 (case 27: acute eosinophilic pneumonia).

## Abbreviations

AI: artificial intelligence
LLM: large language model
Mini-CEX: Mini-Clinical Evaluation Exercise

## Data Availability

The datasets generated or analyzed during this study are available from the corresponding author on reasonable request.
