# Supplementary material for: Quality Assessment of Large Language Model–Generated Medical Dialogue for Clinical Vignettes: Evaluation Study
Source: JMIR Form Res. 2025 Nov 3;9:e80752. doi: 10.2196/80752 (PMC12624296; doi:10.2196/80752)
Supplement: Multimedia Appendix 1 [file formative_v9i1e80752_app1.docx]

Supplement 1. Dialogue with a perfect average score (Case 21: migraine)

1.医師:今日はどうされましたか？

2.患者:ここ数日、すごくひどい頭痛が続いています。

3.医師:それは頭のどの辺りが痛みますか？

4.患者:右側が特に痛いです。こめかみのあたりです。

5.医師:痛みはどのくらいの期間続いていますか？

6.患者:2～3日くらい前からです。最初は軽かったんですが、どんどん強くなってきました。

7.医師:痛みの持続時間はどれくらいですか？ずっと続いていますか？

8.患者:いや、ずっとではなくて、数時間痛くなって、少し良くなってという感じです。

9.医師:頭痛が出るとき、ほかに何か症状はありますか？

10.患者:吐き気がしたり、光がまぶしく感じることがあります。

11.医師:音やにおいにも敏感になったりしますか？

12.患者:はい、音がうるさく感じたり、家族が作る料理の匂いも気になります。

13.医師:頭痛が始まる前に、何か前兆のようなものを感じたことはありますか？

14.患者:そうですね、痛みが始まる前に視界がチカチカしたり、ギザギザした光が見えることがあります。

15.医師:その視界の異常は、いつも何分くらい続きますか？

16.患者:10分から20分くらいですかね。

17.医師:これまでに同じような頭痛を経験されたことはありますか？

18.患者:はい、今までに何度かありました。月に1回くらいです。

19.医師:初めてそういった頭痛を感じたのはいつ頃ですか？

20.患者:10代くらいの頃だったと思います。学生のときによくありました。

21.医師:頭痛がひどいとき、普段できることが何かできなくなったりしますか？

22.患者:そうですね、ひどいときは仕事に行けなかったり、家事も全然手につきません。

23.医師:頭痛が良くなるきっかけとか、逆に悪化するきっかけはありますか？

24.患者:寝不足やストレスが重なるとひどくなる気がします。逆に休むと少し良くなることが多いですね。

25.医師:ご家族の中に、同じような頭痛を経験されている方はいらっしゃいますか？

26.患者:母が似たような症状を訴えていたことがあります。

27.医師:最近の生活で、何か特別変わったことや大きなストレスはありましたか？

28.患者:仕事が忙しくて、いつも以上に疲れている感じはします。

29.医師:これまでに、この頭痛で病院を受診されたことはありますか？

30.患者:いいえ、我慢すれば治ると思っていました。

31.医師:市販薬などで対処されましたか？

32.患者:はい、痛み止めを飲むことはありますが、効かないことの方が多いです。

33.医師:頭痛がないときは、特に体調に問題はありませんか？

34.患者:頭痛以外は特にないですね。普段と変わりません。

35.医師:発熱や体の麻痺、目の痛みなどはありませんか？

36.患者:いいえ、そのような症状はないです。

37.医師:頭をぶつけたり、何か怪我をしたことはありませんか？

38.患者:特に思い当たることはないです。

39.医師:視力の低下や物が二重に見えることはありましたか？

40.患者:いえ、視力の変化は感じていません。

41.医師:食欲や睡眠には変化はありましたか？

42.患者:疲れすぎて食欲が落ちたり、寝つきが悪いことはあります。

43.医師:頭痛がひどいとき、薬以外で何か対処していますか？

44.患者:暗い部屋で休むと少し良くなる気がします。

45.医師:お酒やカフェインを飲むと頭痛が悪化することはありますか？

46.患者:お酒はあまり飲まないんですが、コーヒーを飲むと悪化する感じはあります。

47.医師:お話を伺う限り、片頭痛の可能性が高いと思います。

48.患者:そうなんですね、片頭痛って何か特別な治療が必要ですか？

49.医師:治療には適切な薬があります。例えば、急性期にはトリプタン系の薬、SumatriptanやRizatriptanなどを使用します。

50.医師:例えば、Rizatriptan 10mgを症状が出たときに内服することで改善が期待できます。
