# Supplementary material for: Quality Assessment of Large Language Model–Generated Medical Dialogue for Clinical Vignettes: Evaluation Study
Source: JMIR Form Res. 2025 Nov 3;9:e80752. doi: 10.2196/80752 (PMC12624296; doi:10.2196/80752)
Supplement: Multimedia Appendix 2 [file formative_v9i1e80752_app2.docx]

Supplement 2 .Dialogue with a lower average score of 4 (Case 27: Acute eosinophilic pneumonia)

1.医師:今日はどうされましたか？

2.患者:息切れがひどくて、咳も止まらないんです。ここ数日で急に悪くなった気がします。

3.医師:それはつらいですね。咳はどのくらい続いていますか？

4.患者:3日くらい前からですね。最初は軽い咳だったんですけど、だんだんひどくなった感じです。

5.医師:咳には痰も伴いますか？色や量はどうですか？

6.患者:痰はほとんど出ないんです。ただ、咳をするたびに胸が痛くて苦しいです。

7.医師:胸の痛みはどのあたりですか？また、いつから感じていますか？

8.患者:胸全体が締め付けられるような感じです。咳がひどくなった頃からですね。

9.医師:熱は出ましたか？もし出ているなら、いつからですか？

10.患者:出ました。2日前からだと思いますが、昨晩はけっこう高かった気がします。

11.医師:熱はどれくらいの高さかわかりますか？

12.患者:体温計で測ったら38.5度くらいありました。

13.医師:最近、新しい環境に行ったり、新しいことを始めたりしましたか？

14.患者:そういえば、最近職場でタバコを吸い始めたんです。4、5日前くらいからです。

15.医師:以前もタバコを吸っていましたか、それとも今回が初めてですか？

16.患者:初めてです。友人に勧められて吸ったタイミングですね。

17.医師:タバコを吸ってから症状が出始めたんですか？

18.患者:そうですね、吸うようになった直後から、なんとなく体調が悪い感じがしていました。

19.医師:他に、目のかゆみや皮膚に赤い斑点など、アレルギー症状はありませんか？

20.患者:特にないと思います。

21.医師:息切れとのことでしたが、息苦しさはいつが一番ひどいですか？

22.患者:動いた時が一番つらいです。座ってると少し楽になります。

23.医師:寝るときにも息苦しさがありますか？

24.患者:はい、横になると少し息が詰まる感じがします。

25.医師:喉や鼻の症状、例えば鼻づまりや喉の痛みはありますか？

26.患者:はい、喉が少し痛い気がします。でも鼻はいつも通りです。

27.医師:風邪に似た症状があるということですね。他に筋肉痛や関節の痛みはありますか？

28.患者:全体的にだるくて、体も少し痛い感じがします。

29.医師:最近、海外や特定の地域に旅行に行かれたことはありますか？

30.患者:いいえ、ずっと地元にいました。

31.医師:仕事の内容を伺ってもいいですか？

32.患者:建設業です。前はデスクワークだったんですが、最近現場作業を始めました。

33.医師:現場では何か吸い込むようなものありますか？例えば煙や粉塵など。

34.患者:たまに煙っぽいものを吸い込むことはありますね。でもそんなに頻繁ではないです。

35.医師:ちなみに、ご家族に同じような症状の方はいらっしゃいませんか？

36.患者家族:いいえ、家族はみんな元気です。

37.医師:以前に喘息や肺炎のような病気をしたことはありますか？

38.患者:いえ、特に覚えはないです。

39.医師:肺や気道の病気の診断を今までに受けたことはないですか？

40.患者:はい、何も言われたことはないです。

41.医師:新しく服用されている薬や健康食品などはありませんか？

42.患者:特に何も飲んでいません。

43.医師:では吸入薬やスプレーのようなものは使っていますか？

44.患者:いえ、何も使っていません。

45.医師:レントゲンと血液検査の結果を見させていただきました。胸のCTを参考にもう少し詳しい検査をさせてください。

46.患者:わかりました。それで原因がわかるんですか？

47.医師:可能性があります。CTで肺に影が見え、血液検査では好酸球という細胞が増加していました。

48.医師:急性好酸球性肺炎という病気の可能性が高いです。これはタバコやアレルギー物質への反応が原因で起こることが多いです。

49.患者:そうなんですか...治るんでしょうか？

50.医師:治療にはステロイド、特にPrednisoloneを使用します。初回は20~40mg/日から始めます。これで症状が改善しやすいです。
